# Non-Invasive BCI-VR Applied Protocols as Intervention Paradigms on School-Aged Subjects with ASD: A Systematic Review

**DOI:** 10.3390/s25051342

**Published:** 2025-02-22

**Authors:** Archondoula Alexopoulou, Pantelis Pergantis, Constantinos Koutsojannis, Vassilios Triantafillou, Athanasios Drigas

**Affiliations:** 1 Department of Electrical and Computer Engineering, University of Patras, 26334 Patras, Greece; 2 Net Media Lab & Mind & Brain R&D, Institute of Informatics & Telecommunications, National Centre of Scientific Research ‘Demokritos’, 15341 Agia Paraskevi, Greece; pantperg@helit.duth.gr; 3 Department of Information & Communication Systems Engineering, University of the Aegean, 83200 Karlovasi, Greece; 4 Health Physics & Computational Intelligence Lab, Physiotherapy Department, School of Health Rehabilitation Sciences, University of Patras, 25100 Patras, Greece; ckoutsog@upatras.gr; 5 Network Technologies and Digital Transformation Lab, Electrical and Computer Engineering Department, University of Peloponnese, 26504 Patras, Greece; vtriantaf@uop.gr

**Keywords:** brain–computer interface (BCI), virtual reality (VR), autism spectrum disorder (ASD), neurofeedback (NFT), EEG signals, social skills, joint attention, memory, emotional regulation

## Abstract

This paper aims to highlight non-invasive BCI-VR applied protocols as intervention paradigms on school-aged subjects with ASD. Computer-based interventions are considered appropriate for users with ASD as concentration on a screen reduces other stimuli from the environment that are likely to be distracting or disruptive. Since there are no social conditions for engagement in such processes and the responses of computing systems do not hold surprises for users, as the outputs are fully controlled, they are ideal for ASD subjects. Children and adolescents with ASD, when supported by BCI interventions through virtual reality applications, especially appear to show significant improvements in core symptoms, such as cognitive and social deficits, regardless of their age or IQ. We examined nine protocols applied from 2016 to 2023, focusing on the BCI paradigms, the procedure, and the outcomes. Our study is non-exhaustive but representative of the state of the art in the field. As concluded by the research, BCI-VR applied protocols have no side effects and are rather easy to handle and maintain, and despite the fact that there are research limitations, they hold promise as a tool for improving social and cognitive skills in school-aged individuals with ASD.

## 1. Introduction

This paper presents some of the most representative studies and applied protocols in the field of brain–computer interfaces, delivered through virtual reality environments to children and adolescents with autism spectrum disorder. The aim is to ascertain whether VR protocols are delivered efficiently through BCI systems in order to improve core ASD symptoms. Autism is a neurodevelopmental disorder of multifactorial aetiology mainly characterised by deficiency in social and language skills.

Neurodevelopmental disorders are cognitive and behavioural disorders that occur throughout a person’s development period, characterised by difficulties acquiring and performing specific cognitive, motor, or social skills and functions. Their aetiology is complex and unknown in many cases as indicated by the American Psychiatric Association [1]. Accordingly, a variety of constrained and repetitive patterns of behaviour and interests, as well as ongoing deficiencies in the capacity to initiate and sustain two-way communication and social engagement, are characteristics of autism spectrum disorder [2]. The clinical symptomatology does not diminish with age but is present for the majority of young and adult individuals for the rest of their lives, with variations in intensity, mode of manifestation, and functioning [3,4].

Interventions aim to improve the quality of their life, reducing the occurrence of symptoms while increasing their functionality, as the acquisition of a degree of autonomy and social skills is considered essential.

### 1.1. BCI Technology

A human brain–computer interface system is a tool that enables communication with others without the need for vocal or physical touch [5]. It works by converting brain activity into commands for a computer system via signal processing techniques. By monitoring the voltage variations in the ionic influx into the neurons, EEG captures the electrical activity generated by the various brain areas. The computer system then shows the user’s thoughts in real time on a screen. It is a direct communication channel between an external device and the brain of an individual who has deficiencies in cognitive and social abilities or sensorimotor processes, avoiding the typical sensory or motor pathways [6]. Each group that receives a BCI has a neurological impairment or deficiency [7,8].

According to Wolpaw and Wolpaw [9], this technology records brain activity in different brain regions, processes it, and outputs an artificial signal that may enhance, support, replace, or otherwise improve the regular functioning of the central nervous system. An individual can be impacted either internally—by his body—or externally—by his interactions with the world through the use of a brain–computer interface system, which records brain signals, extracts their properties, and converts them into artificial effects.

**Figure 1:**
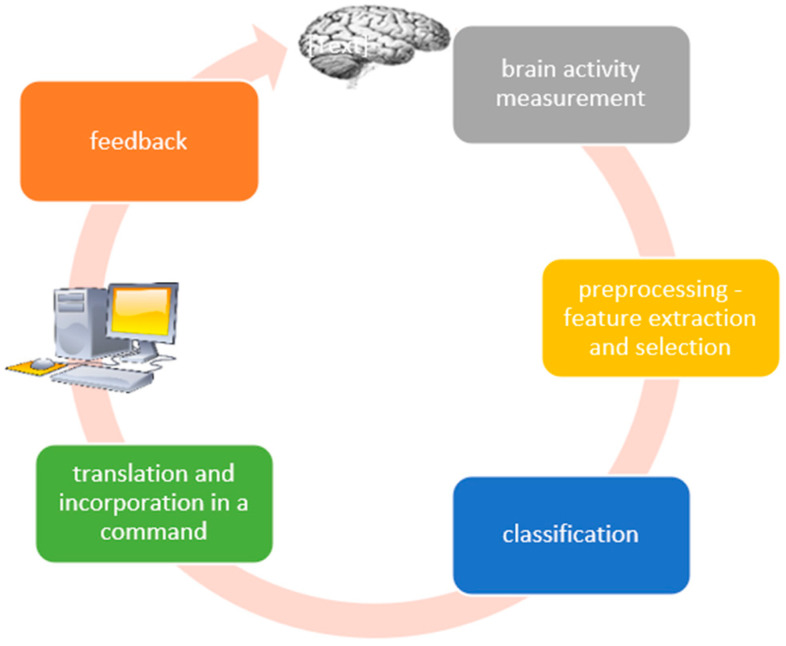
A typical BCI system.

Figure 1 illustrates how a closed feedback loop system [10,11] functions, following a predetermined procedure common to all kinds of brain–computer interface (BCI) systems. The six main steps in this process are as follows: brain activity measurement, which records neural signals; preprocessing, which removes noise or artefacts that might skew the signal; feature extraction, which finds pertinent patterns in the data; classification, which deciphers these patterns to ascertain the user’s intent; translation and integration, which transforms the intent into a specific command; and feedback, where the system gives the user real-time responses to help and inform them during the interaction.

**Figure 2:**
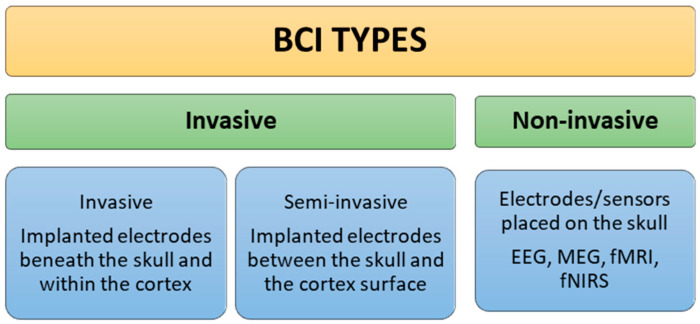
BCI types.

Finally, based on their interactions with the brain, brain–computer interfaces (BCIs) can be divided into invasive and non-invasive categories, as shown in Figure 2. High-resolution signals and exact control are provided by invasive BCIs, which include the surgical insertion of electrodes straight into the brain. These are frequently employed in medicine to help people who are paralysed regain their range of motion.

Non-invasive BCIs, on the other hand, use external tools to monitor brain activity, like near-infrared spectroscopy (NIRS) or electroencephalography (EEG) caps [7]. They are safer and easier to use, which makes them appropriate for uses like gaming, communication aids, and neurofeedback therapy, even though their precision can be diminished by signal interference from the skull and scalp.

The protocols used for people with ASD are non-invasive and safe to use, since no side-effects have been mentioned during their application. Our aim is to shed light on the current research and verify the positive outcomes in subjects of school age with ASD.

### 1.2. BCI Technology and ASD

Autism spectrum disorder is a difficult area of intervention given the multifactorial nature of its origin, evolution, and manifestation and also the presence of several comorbidities. Nevertheless, interventions with modern technological means are constantly gaining ground in the last two decades as alternative or complementary forms of intervention [12,13]. Children with ASD, when supported by computer-based interventions and virtual reality applications, especially appear to show significant improvements in autistic symptomatology, regardless of their age or IQ [14,15].

A determining factor contributing to the successful use of human brain–computer interface technologies in ASD is the nature of the disorder itself, as people with ASD prefer a stable environment around them with as little stimulation and change as possible [16]. That is what the specific intervention systems provide since little or no interaction with other people is required, as even the instructions for the procedure are provided visually and/or audibly through a neutral and blank tone of voice [17]. Another advantage of using BCI technologies, according to Tseng and Do [18], is that they can provide education and support to people with autism throughout their lives, relieving their families and themselves from the heavy financial burdens of other forms of intervention.

Consequently, brain–computer interfaces (BCIs) are cutting-edge resources for helping people with ASD. These devices are able to track brain activity in order to detect distinct neural patterns linked to difficulties, including sensory processing problems, emotional dysregulation, or concentration deficiencies. In order to assist people to control their brain activity and acquire important abilities, BCIs can then offer interventions, like neurofeedback training. Furthermore, when paired with interactive technologies like virtual or augmented reality, BCIs develop secure environments for practicing coping mechanisms or social interactions that are customised for each person.

### 1.3. VR Systems in ASD

Virtual reality is a combination of technologies that enable both the creation and the navigation through virtual environments, which are 3D digital representations of realistic ones. The human senses perceive these representations in the same way as they perceive the real world [19]. The user can navigate between them and engage in real-time interaction with virtual objects or characters, as their final appearance closely resembles real-life situations [20]. The main goal for the users on the autism spectrum has always been to develop social–communication skills through scenarios taking place in ideal-for-autism virtual environments.

In other words, the application of virtual reality to autism comprises the imitation of ideal, realistic environments through simplification to a level tolerated by the user, with the integration of visual and audio sensory information. The scientific results of this application have been particularly encouraging since it has been observed that users on the autistic spectrum are able not only to monitor themselves on a screen but also process the specific virtual reality scenarios successfully [15,20,21,22,23,24,25]. This happens because virtual reality intervention protocols in autism have the advantage of complete control of complex stimuli, providing opportunities for personalised intervention within a virtual, safe, and well-structured environment [26]. The ultimate goal is to produce improved attitudes, anticipations, and generalisations about the real world by using such interventions.

Unlike more traditional models of interventions, virtual reality systems represent everyday experiences in a controlled, stress-free, and inclusive environment, allowing for extensive exposure and repetitive practice [24,25]. At the same time, learning communicative behaviours can ultimately lead to greater flexibility in their social lives [15,27]. This is achieved due to the diversity of the virtual scenarios and the representation of different social stimuli and situations through them [24]. Moreover, there is the possibility of shaping the virtual characters in such a way as to stimulate the learner’s environment, even by providing the distinctive characteristics of a race [28] so as to achieve a significant degree of comfort and appropriation. This flexibility and diversity, combined with the absence of potentially stressful factors, make virtual reality education platforms an effective way to develop and enhance the social skills of people on the autism spectrum [25].

Additionally, more complex forms of virtual reality such as augmented virtual reality, where virtual reality is projected over the real world, and immersive virtual reality, where the user has direct first-person interaction with the digital environment, have already been developed on mobile devices as parts of intervention protocols. After the implementation of the augmented reality systems in such protocols, there have been several positive outcomes as well.

Among the others, the development of interactive behaviours is noticeably enhanced in these environments while at the same time the CNS is activated, and significant cognitive and motor improvements can occur due to neuroplasticity [29]. The augmented reality systems can also be used to train children with ASD to adapt in new environments or everyday situations, without creating stress for them [30].

On the other hand, in virtual reality immersion systems, the users integrate themselves as often as they wish in a world created with three-dimensional representations of a realistic environment. This environment provides the user with specific visual information, something that corresponds to the mechanism of visual perception and information absorption of individuals on the autism spectrum [31]. This technology has already been used efficiently to cultivate social skills [32,33] and develop and improve the emotional response [31] in children and adolescents with ASD.

Technically speaking, BCI-VR systems are primarily classified based on the BCI paradigms they employ: motor imagery (MI) potential; event-related potential (ERP), such as P300; and steady-state visual evoked potentials (SSVEPs) [5,7,9,11]. Each of these paradigms, displayed below, leverages unique neural responses to interface with virtual reality environments.

Motor Imagery (MI) Potential: This paradigm relies on the neural activity generated when a user imagines moving specific body parts, such as a hand or foot. These imagined movements produce measurable brain patterns known as event-related desynchronisation (ERD) and event-related synchronisation (ERS), which can be decoded to control VR systems. MI-based BCIs are particularly suited for rehabilitation and motor skill training applications.

Event-Related Potential (ERP): A common example of ERP is the P300 component, which is a positive deflection in the EEG signal occurring approximately 300 milliseconds after a user perceives an infrequent or significant stimulus (known as the oddball paradigm). The P300 signal is often used to identify targets or selections that a user focuses on, making it an effective paradigm for applications, like communication systems or virtual object selection in VR.

Steady-State Visual Evoked Potentials (SSVEPs): This paradigm capitalises on the brain’s response to visual stimuli that flicker at specific frequencies. When a user gazes at a stimulus, the visual cortex generates signals that match the flickering frequency, which can be detected and used to control VR elements. The excellent accuracy and dependability of SSVEPs make it appropriate for immediate operation and navigation in virtual settings.

These three BCI paradigms have been extensively researched and developed, leading to their widespread adoption in real-world applications. They enable intuitive and adaptive interactions with virtual reality, opening new possibilities in fields such as gaming, rehabilitation, education, and assistive technologies. Moreover, this technology holds promise for fostering greater autonomy and improving core symptoms for people with ASD.

## 2. Materials and Methods

### 2.1. Study Design

To ensure rigorous and robust delivery through the identification, selection, assessment, and synthesis of the finalised included studies, the study design of this research was derived from the application of the systematic review approach, followed by the PRISMA statement of systematic reviews and meta-analyses. This systematic review protocol was registered on the Open Science Framework (accessed on 22 January 2025 https://osf.io/6x43g/).

The PRISMA principles also established a comprehensive and transparent methodology in our systematic review on the use of brain–computer interfaces (BCIs) and virtual reality (VR) to ameliorate core symptoms in ASD. This method improved the reproducibility, dependability, and trustworthiness of our findings by offering a strict structure for the identification, selection, and critical assessment of pertinent research studies. With regard to the final chosen studies that were examined, this systematic review aims to address the following research questions.

**RQ1.**  *Is BCI-VR technology able to improve ASD core symptoms?*

**RQ2.**  *Are these interventions applicable in the real world?*

**RQ3.**  *Which BCI-VR settings were employed?*

### 2.2. Inclusion and Exclusion Criteria

We established precise inclusion and exclusion criteria in order to create a solid and thorough assessment of the research:

#### 2.2.1. Inclusion Criteria

- Research involving participants aged 5–20 diagnosed with ASD and assessed with valid rating scales, psychometric tools, and/or human assessment.
- Research employing VR and non-invasive BCI interventions specifically aimed at regulating ASD core symptoms.
- Articles that investigated emotional regulation and memory, social, and attention skills in subjects with ASD, delivered through VR-BCI systems.
- Publications that were presented at prominent academic conferences or published in peer-reviewed journals.

#### 2.2.2. Exclusion Criteria

- Articles that presented research on VR and ASD without explicit or implicit use of BCIs. Research that presented the non-invasive use of BCI technology.
- Editorials and columns of opinion were not included, nor were theoretical papers with no empirical support.
- Research conducted for commercial reasons was excluded.
- Papers not written in English were also excluded

### 2.3. Search Strategy and Information Sources

A thorough and methodical search was carried out to find pertinent studies published up until March 2024 across a number of significant electronic databases, including IEEE Xplore, Web of Science, PubMed, Scopus, and, as a complement, Google Scholar. In order to capture the junction of ASD, VR, and BCIs, a comprehensive list of keywords and concepts was used in the search approach. The following particular search phrases and combinations were used:

“ASD” AND “virtual reality”.
“EEG” AND “ASD”.
“ASD” AND “EEG interventions”.
“ASD” AND “brain-computer interface”.
“VR” AND “autism treatment”.
“Non-invasive BCI” AND “autism treatment”.
“BCI” AND “VR” AND “ASD”.
“Virtual reality” AND “neurofeedback”.
“Brain-computer interface” AND “autism treatment”.

These phrases were efficiently combined using Boolean operators, which improved the precision and scope of the search. In order to guarantee a high degree of scientific quality and relevance, database-specific filters were also used to restrict the results to peer-reviewed journal articles and conference papers published in English.

### 2.4. Study Selection

In order to find research according to the inclusion requirements, two impartial reviewers went through the titles and abstracts of every publication that our search yielded. A full-text review of possibly pertinent studies was conducted after the initial screening. Discussion and consensus were used to settle any disagreements among the reviewers regarding paper inclusion and exclusion. To maintain objectivity during the selection process, a third reviewer was consulted if differences continued.

### 2.5. Data Extraction

A standardised form created to gather important information from every included study was used to extract data. Among the important data points that were extracted were the following:

Study Design: This included the kind of research under study and clarifying how solid it was.
Participant Characteristics: The population under investigation and the generalisability of the results were defined by recording data, such as sample size, demographic information like age and gender, and participant diagnoses and/or comorbidities.
Intervention Details: The type of VR settings, the length and frequency of the sessions, and the particulars of the BCI paradigms were all noted, along with thorough information about the VR and BCI interventions.
Outcome Measures: We recorded the findings of the NFT sessions together with the EEG locations used by the research teams. This aided in appreciating the effectiveness and significance of the interventions.
Results: The main results were documented, focusing on the reported effects on social skills, attention, and emotional regulation.
Methodological Quality: Additionally, an evaluation of the possible biases of the studies and the diligence in methodology was carried out.

### 2.6. Included Studies

A total of *n* = 645 articles were located and screened for the final selection of the included articles after applying processing, inclusion, and representative criteria for the main part (*n* = 9) in the final selection for further study and analysis. The first step in the selection process was the establishment of the eligibility criteria. In order to maximise the number of results, a list of keywords was also generated to initiate the database search, which used a range of search filters and Boolean operators. The selection process was carried out using title and abstract screening in accordance with the eligibility criteria (*n* = 317) after duplicate studies (*n* = 328) were eliminated.

**Figure 3:**
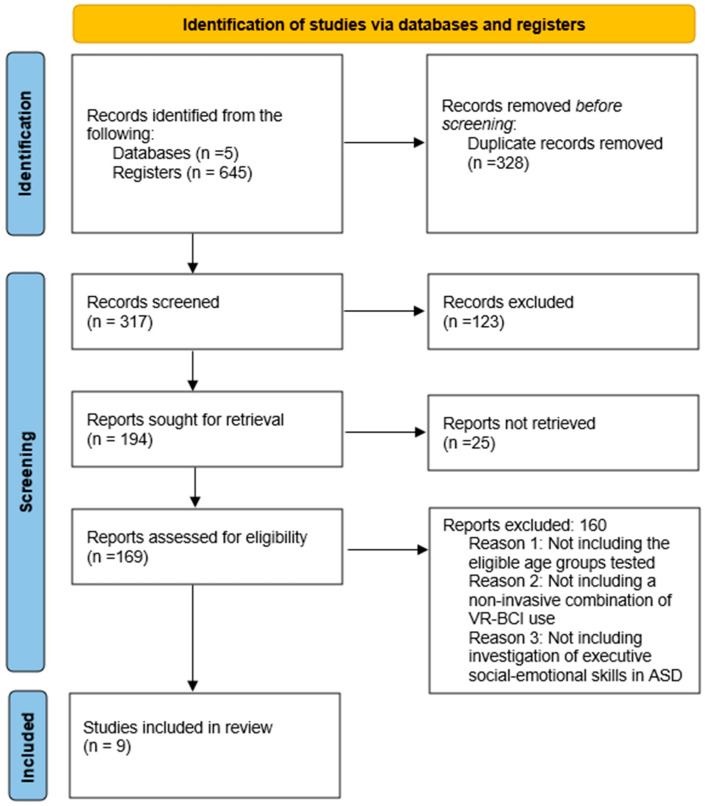
PRISMA Flow Diagram.

The next step in the process was full-text screening after *n* = 123 publications were excluded. The remaining studies (*n* = 194) were processed thoroughly. Regretfully, retrieving *n* = 25 articles for full-text screening was not feasible. The final selection process involved three independent reviewers (C.K, V.T, and A.D) who debated whether the eligibility requirements were applicable while looking over the full texts of *n* = 38 papers. The remaining *n* = 160 were removed since they did not fit the eligibility criteria. Consequently, *n* = 9 items were included in the final selection (Figure 3).

### 2.7. Quality Assessment

The listed studies’ methodological quality was evaluated using established tools. Randomised controlled trials were conducted using the Cochrane Risk of Bias tool, which assesses selection, blinding, allocation concealment, random sequence generation, and inadequate outcome data. Cohort comparability, exposure or outcome determination, and case selection quality were evaluated in observational studies using the Newcastle–Ottawa Scale.

### 2.8. Synthesis of Results

During the synthesis process, both qualitative and quantitative analyses were carried out. We compiled the main conclusions qualitatively, placing emphasis on recurring trends, perspectives, and possible research inadequacies. For the quantitative synthesis part, effect sizes indicative of the intervention effects were estimated for similar outcomes across several trials.

Overall, this thorough methodological approach ensured our systematic review was trustworthy and consistent, laying the ground for further studies and applications in VR, BCIs, and ASD.

## 3. Results

### 3.1. Studies’ Characteristics

**Figure 4:**
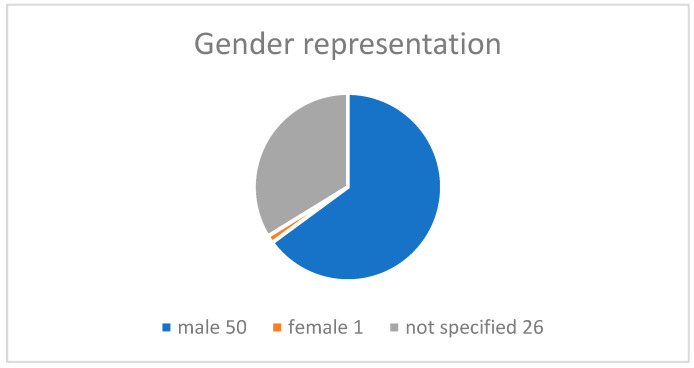
Gender representation.

**Figure 5:**
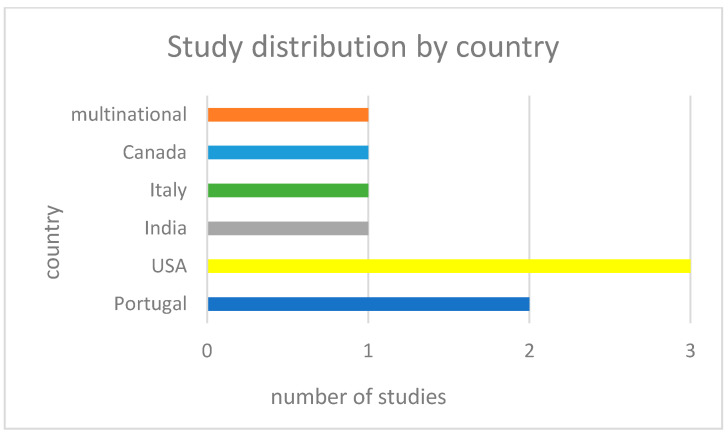
Study distribution by country.

**Figure 6:**
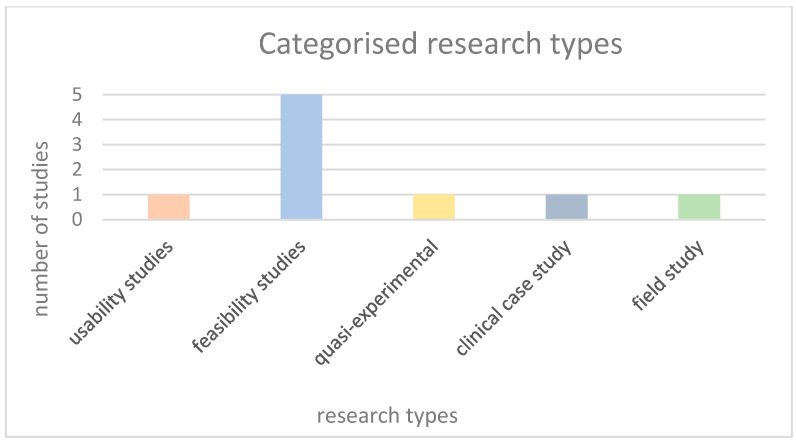
Categorised research types.

The final study selection concerned children and adolescents of school age, including some pre-schoolers in one case and some others close to twenty in another. A total of *n* = 77 target group participants were analysed. The control group consisted of *n* = 9 participants, indicating the difficulty in recruiting ASD youngsters to take part in this kind of research. Specifically, out of nine studies, only two had control groups. The mean age of the participants was *n* ≈ 12.59. (See Figure 4). According to the participants’ gender, *n* = 50 were males, *n* =1 was female, and *n* = 26 were not gender-specified. The figures indicate the male over female prevalence in ASD. Regarding the country of the study origin, we observed *n* = 2 [34,35] from Portugal, *n* = 3 from the USA [36,37,38], *n* = 1 from India [39], *n* = 1 from Italy [40], *n* = 1 from Canada [41], and *n* = 1 multinational, including the USA and India [42]. According to the study design, the researchers established several different kinds of methodologies. These include quasi-experimental designs [42], feasibility studies [34,37,38,39], usability studies [36], clinical case studies [40] , and field studies [41]. (See Figure 5 and Figure 6). Additionally, the treatment results were assessed either by psychometric tools like SCQ, SRS, NEPSY [36], POMS, JAAT, ATEC, VABS [35], CARS [39], CGI, ADOS2, LEITER-R, and CARS2-ST [40] or by expert therapists and/or parents/caregivers, as in [36,37,38,41]. Finally, in two studies [34,42], the type of assessment was not specified.

### 3.2. RQ1. Is BCI-VR Technology Able to Improve ASD Core Symptoms?

Recent advancements in the integration of VR with BCIs have shown substantial promise in addressing the core symptoms of autism spectrum disorder (ASD). These innovative paradigms leverage neuroplasticity—the brain’s ability to reorganise itself—to create targeted interventions for individuals with ASD. Below is a detailed expansion on the reported findings:

#### 3.2.1. Social Deficits

BCI-VR systems provide immersive and controlled virtual environments that can significantly benefit individuals with autism spectrum disorder (ASD) in addressing social deficits. These systems create a safe space where users can practice and develop essential social skills without the pressure of real-world interactions.

Eye Contact: One of the key challenges for individuals with ASD is making and maintaining eye contact during conversations [36]. BCI-VR systems can simulate virtual characters that encourage and reward appropriate gaze behaviour [34,35]. By providing a virtual character that responds positively to direct eye contact, the system helps individuals gradually become more comfortable with this social cue, reinforcing eye contact as a social norm.

Conversational Skills: Communication can often be difficult for those with ASD, particularly when it comes to initiating or sustaining conversations [36,41]. BCI-VR systems can simulate various conversational scenarios with virtual characters, allowing users to practice these skills in a low-pressure environment. Users can engage in dialogues where they take turns speaking, practice appropriate responses, and learn how to navigate social exchanges [36]. This reduces the anxiety associated with real-world conversations, as users become more familiar with the flow and expectations of verbal interactions.

Feedback Mechanisms: Real-time feedback is crucial for helping individuals with ASD adjust their social behaviours. BCI-VR systems can integrate feedback tools such as head orientation and gaze tracking [34,35,41] to give users immediate information about their social attention. For example, the system might provide visual or auditory cues if a user is not making eye contact or facing the virtual character appropriately. By receiving this type of feedback, users can quickly adjust their behaviour and learn to interpret social cues more effectively, improving their ability to interact with others in various social settings.

#### 3.2.2. Emotional Regulation

VR-BCI systems seem to be effective tools for helping individuals with ASD to improve their emotional identification, understanding, and regulation. These systems may offer tailored and interactive experiences that address the unique challenges associated with emotional awareness and control.

Simulated Emotional Scenarios: VR-BCI systems can immerse users in simulated environments designed to teach emotional regulation and expression [34,35,36,38]. Additionally, scenarios can be individually designed to help users practice recognising and expressing appropriate emotions during interactions with virtual characters.

#### 3.2.3. Sensory Awareness

Individuals with autism spectrum disorder (ASD) frequently encounter difficulties with sensory processing, which can lead to heightened sensitivity or sensory overload in everyday environments. VR-BCI systems may offer a structured and supportive platform to address these challenges through gradual and controlled exposure to sensory stimuli.

Controlled Sensory Exposure: All VR environments under examination offer simulation of a wide range of sensory experiences, such as different sounds, lights, or visual patterns, allowing for the careful and individualised introduction of these stimuli. This controlled exposure is tailored to the user’s tolerance levels, reducing anxiety and helping them build resilience.

Reduction in Sensory Overload: Over time, exposure to these tailored VR experiences can enhance sensory integration and awareness, helping individuals with ASD become less overwhelmed by sensory input in their everyday lives [35,36,37,40,41]. This can lead to improved comfort and functionality in environments, such as schools, workplaces, or social settings.

#### 3.2.4. Attention Training

VR-BCI protocols have proven effective in enhancing both general attention and joint attention, which are crucial skills for social communication and interaction. These systems leverage engaging and interactive environments to strengthen these cognitive abilities in individuals with ASD.

Enhancing General Attention: VR-BCI systems often incorporate gamified training modules that require users to concentrate on specific tasks or track visual and auditory stimuli. For example, a user might need to follow a moving object, respond to specific cues, or solve puzzles within the virtual environment [35,40,41]. These activities not only keep users engaged but also improve their attentional control and focus, skills that are essential for learning and everyday functioning.

Improving Joint Attention: Joint attention, or the ability to share focus on an object or event with another person, is a key component of social communication that is often challenging for individuals with ASD [43]. VR-BCI systems simulate interactive scenarios that encourage users to practice joint attention, such as responding to a virtual character pointing to an object or following a shared focus during a game [34,35]. This targeted practice seems to help users develop the ability to coordinate attention with others, a skill that is foundational for building social connections and participating in collaborative activities.

#### 3.2.5. Memory Improvement

Engaging and interactive VR-BCI tasks have the potential to significantly strengthen both working memory and long-term memory by offering stimulating, immersive, and repetitive practice opportunities tailored to individual needs.

Improving Working Memory: VR-BCI tasks often require users to remember and execute sequences of actions within virtual environments, such as following a series of steps to solve a puzzle or navigate a simulated space. These activities challenge users to temporarily store and manipulate information, strengthening their working memory capacity [42].

Enhancing Long-Term Memory: By integrating learned information into simulated real-life scenarios, VR-BCI systems help users practice retrieving and applying knowledge in meaningful contexts. These immersive experiences reinforce long-term memory by linking knowledge to practical applications, increasing the likelihood of retention.

#### 3.2.6. Cognitive Development

Respondents demonstrated improved cognitive states, which can be attributed to the highly stimulating and engaging nature of VR-BCI activities. These systems offer dynamic, interactive experiences that encourage active participation and cognitive growth.

Fostering Cognitive Engagement and Development: VR-BCI activities, such as problem-solving tasks or decision-making challenges, create an immersive environment where users are motivated to focus and think critically [35,41,42]. These tasks promote mental flexibility and a readiness to absorb and process information effectively.

Nevertheless, the examined research has a number of limitations, which will be covered in detail in Section 5. Concluding the results emerging from our systematic review, this promising convergence of VR and BCI technologies offers new hope for individuals with ASD, providing tools to enhance their quality of life and independence.

### 3.3. RQ2. Are These Interventions Applicable in the Real World?

The primary aim of VR-BCI interventions is to facilitate the transfer of skills learned in virtual environments to real-world scenarios, empowering individuals with ASD with practical abilities that improve their daily lives. These interventions are designed to bridge the gap between virtual practice and real-life application in meaningful and measurable ways. This systematic review has yielded the following results:

Perspective-Taking: VR-BCI systems provide immersive experiences that allow users to “step into someone else’s shoes”. This fosters empathy, improves social understanding, and supports the development of advanced social skills critical for building and maintaining relationships for ASD subjects [35,36].

Daily Interactions: Skills honed in VR, such as teamwork or navigating public spaces, translate directly to real-world contexts. By practicing these scenarios in a controlled, low-pressure virtual environment, ASD users can gain confidence and competence, leading to improved interactions and independence in everyday life [36,39,40,41].

Driving Simulation: VR-BCI interventions can also include driving simulations that help users develop confidence and practical skills for safe driving. These scenarios replicate real-world driving conditions, allowing users to practice vehicle control, traffic navigation, and situational awareness. For individuals with ASD, mastering these skills enhances their independence and mobility [37,38].

### 3.4. RQ3. Which BCI-VR Settings Were Employed?

The brain–computer interface (BCI) protocols examined in the first group of studies focus on using virtual reality (VR) and EEG-based monitoring to enhance cognitive, social, and motor skills in individuals with autism spectrum disorder (ASD). These studies integrate real-time physiological and neural data to assess engagement, workload, and learning patterns.

In particular, the following is a brief description of the five BCI protocols that share several characteristics:

EEG Recording Systems:

- The Emotiv EPOC 14-channel headset (Emotiv Inc., San Francisco, CA USA)was used in all the studies [36,37,38,39,42].
- Electrodes were placed at AF3, AF4, F3, F4, F7, F8, FC5, FC6, T7, T8, P7, P8, O1, and O2 following the 10–20 system.
- EEG signals were sampled at 128 Hz, with a 0.2–45 Hz bandwidth, focusing on the alpha, beta, gamma, theta, and delta frequency bands.
- VR Environment and Simulators:
- A VR-based driving simulator used a Logitech G27 Controller (Logitech International S.A., Lausanne, Switzerland)with steering and pedals [37].
- VR learning environments involved interactive tasks, such as classroom-based education and social interaction training [36,39].
- VR scenarios were created using CityEngine (Esri R&D Center Zurich, Switzerland), Autodesk Maya (Autodesk, Inc., San Francisco, CA, USA) and custom-built immersive setups [38].

Gaze and Physiological Data Tracking:

- Tobii eye-tracking devices (Tobii AB, Stockholm, Sweden) measured gaze fixation and visual processing patterns [36,37,38].
- The Biopac MP 150 system (BIOPAC Systems, Inc., Goleta, CA, USA) recorded heart rate, skin conductance, and muscle activity to assess emotional and cognitive responses [38].
- The BioNomadix Physiology system (BIOPAC Systems, Inc., Goleta, CA, USA) was used as a physiological measurement system [36].

Data Analysis and Evaluation:

- EEG data were analysed for functional connectivity, coherence, phase locking, and workload modelling to evaluate cognitive engagement [36,37,38,39,42].
- Expert therapist [37,38] and parent/caregiver [36] evaluations complemented automated data analysis to assess learning progress, engagement, and stress levels.

The second set of studies showed the possibility of adaptable VR interventions by analysing cognitive and emotional responses in people with ASD using various EEG-based BCI settings with VR environments. Each protocol leveraged an EEG-based BCI with immersive or augmented reality to train social attention, focus, and cognitive skills in ASD individuals, showing promising but varied effectiveness. The EEG data analysis [35,40,41] was supplemented by parent and/or caregiver evaluations in [41]. Below is a brief overview of the BCI protocols that were employed:

- BCI Type: P300-based EEG.
- EEG Systems: g.Mobilab+ (g.tec medical engineering GmbH Schieldberg Austria), g.Nautilus, (g.tec medical engineering GmbH Schieldberg Austria), and V-Amp (Brain Products GmbH, Gilching, Germany).
- Electrode Placement: C3, Cz, C4, CPz, P3, Pz, P4, and POz.
- Paradigm: Oddball detection (P300) to track attentional shifts.
- VR Hardware: Head-mounted display in Amaral et al. [34].
- BCI Type: P300-based EEG with eye-tracking.
- EEG System: g.Nautilus (wireless).
- Electrode Placement: C3, C4, P3, and POz (ground: AFz; reference: right ear).
- Paradigm: P300-based neurofeedback for attentional tracking.
- VR Hardware: Oculus Rift DK2 (Oculus VR, CA, USA) with integrated eye-tracking in Amaral et al. [35].
- BCI Type: Steady-state visual evoked potential (SSVEP).
- EEG System: Olimex EEG SMT (Olimex Ltd. Plovdiv, Bulgaria)
- Electrode Placement: FPz and Oz (reference: wrist).
- Paradigm: SSVEP-based visual attention (flashing arrows for control).
- AR Hardware: Moverio BT-200 glasses (Epson, Suwa, Japan).
- Robot: SanBot Elf (Qihan Technology Co., Ltd., Shenzhen, China)—executing movement based on EEG inputs, in Arpaia et al. [40].
- BCI Type: Mu rhythm suppression-based neurofeedback.
- EEG System: BrainCo FocusCalm headband (BrainCo Inc., Somerville, MA, USA)
- Paradigm: Real-time mu rhythm suppression to adjust AR game elements.
- AR Hardware: Tablet with Unity XR API.

Each study utilised different EEG paradigms (P300, SSVEP, and mu suppression) and combined BCI with VR, AR, and robotics to enhance attention and social skills in ASD individuals. See Lyu et al. [41].

## 4. VR-BCI Non-Invasive Protocols in Autism

Brain–computer interface systems are constantly grabbing the interest of the people who deal with virtual reality since this technology has the potential to improve system accuracy, decrease calibration time, and improve user mental state recognition. It offers many possibilities for easy interaction in VR environments, with the aid of wireless technology [10]. The interaction degree between the system and the trainee is determined by both the technological characteristics of the system used and the trainee’s potential [26]. Above all, the main goal still remains the same: to improve their behaviour and develop the emotional and social skills of people with ASD. Indeed, the interaction in a virtual reality environment in which stimuli related to the observation and imitation of actions have been incorporated serves this goal [44].

With these encouraging outcomes in mind, our goal is to examine BCI-VR applications with an emphasis on those that have to do with human cognition and the core symptomatology of young people with ASD. In order to achieve this, we go over non-invasive BCI systems that are based on electroencephalography (EEG), outlining the different brain waves, target electrodes, and neurofeedback training session outcomes.

In particular, we give a summary of BCI systems that use EEG control signals based on steady-state visual evoked potentials (SSVEPs), motor imagery, and the P300 component. These systems relate to the cognitive processes of perception, attention, memory, and social functioning. Furthermore, we go over the targets, methods, and results of VR-BCI protocols and talk about the pertinent research gaps and future research options. The most prominent research on applied VR protocols of the last decade is described below, after the presentation of the summary table with the research papers, their contributions, and their limitations (Table 1).

The presentation in two parts of the research papers included in this review is based on the brain areas selected to be treated, the dysfunction of which results in ASD core symptoms. People with ASD have characteristic dysfunctions in the lobes, the somatosensory cortex, the sensorimotor cortex, and the cingulate gyrus. Since certain brain activity is linked to the EEG frequency bands, the positioning of the electrodes on the scalp aligns with the roles of the cerebral cortex’s four lobes. According to the worldwide 10–20 system, which is the most commonly used electrode placement method, the letters F, P, T, and O stand for the electrodes positioned over the frontal, parietal, temporal, and occipital lobes, respectively. The electrodes with odd numbers are positioned at certain points on the left side of the scalp, while those with even numbers are positioned at specific points on the right side. The midline positions are displayed by z.

Specifically, the first five research papers have a great deal of the BCI protocols implemented in common, since they all incorporated the Emotiv EPOC 14-channel EEG recorder in their VR-BCI systems, and most of them also used an eye tracker. Finally, they all treated the same brain areas, putting the active electrodes at the same locations, as presented in Table 2 below.

The multimodal adaptive social interaction in VR (MASI-VR) protocol emerges as a technically and procedurally innovative approach for studying and addressing emotional and social deficits in children with autism spectrum disorder (ASD) [36]. Its multimodal nature is reflected in two key features. First, the protocol leverages gaze analysis and adaptation through a unique “face enclosure” technique. This involves gradually revealing a face on the screen as the user processes relevant visual patterns, ultimately selecting a targeted emotional expression. Second, the system employs a structured dialogue model with virtual characters, creating an interactive environment reminiscent of a school cafeteria. This dual approach aims to uncover how children with ASD perceive emotions, potentially informing new intervention strategies to enhance their emotional and social abilities.

The MASI-VR system is underpinned by a sophisticated distributed virtual reality framework comprising five interlinked subsystems: The Adaptive Activity Presentation that tailors the tasks to user progress, the interactive framework that facilitates user engagement with the VR environment, the physiological measurement system that records real-time data for analysis using the BioNomadix Physiology system, the EEG signal recording that employs Emotiv EPOC for brain activity monitoring, and finally, the eye-tracking and feedback that utilises the Tobii X120 remote desktop eye tracker for real-time feedback on gaze patterns.

A central supervisory control system orchestrates the integration of these components, ensuring seamless interaction between peripheral devices and the VR presentation layer. During training, EEG data were collected using electrodes positioned at 14 strategic sites (AF3, F7, F3, FC5, T7, P7, O1, O2, P8, T8, FC6, F4, F8, and AF4), alongside eye-tracking and physiological data. This robust dataset was designed to analyse and improve emotional recognition in users.

The study involved 12 high-functioning boys aged 13–17 diagnosed with ASD, split into experimental and control groups (6 participants each). While the control group trained without visual analysis, feedback protocols, or face-activity tasks, the experimental group received the full MASI-VR experience. Over 3–5 laboratory visits, each lasting approximately one hour, the participants underwent training supervised by a therapist.

The results revealed modest yet promising outcomes. The experimental group showed a 3% improvement in eye contact post-training, a change not observed in the control group. While other metrics did not yield statistically significant differences, the research team expressed optimism about the system’s performance and user engagement. They see MASI-VR as a foundational framework for future systems capable of integrating peripheral data interactively to advance interventions for emotional and social challenges in ASD.

This study highlights MASI-VR’s potential to bridge gaps in understanding emotional processing among children with ASD. While the initial results are incremental, they underscore the system’s capacity to evolve into a more comprehensive and impactful tool for emotional and social development.

The protocol planned by Fan et al. [37] focuses on empowering individuals on the autism spectrum by developing skills that enhance independence in daily life, specifically driving. Driving poses unique challenges for people with ASD, including difficulty learning and maintaining driving skills, anticipating hazards, and managing visual focus. Previous research by the team highlighted distinctive gaze patterns in individuals with ASD, who tend to focus more on the vertical axis and further to the right on the horizontal axis compared to neurotypical individuals. This pattern reflects challenges in controlling the entire visual field, compounded by difficulties in processing complex, simultaneous stimuli. To address these challenges, the researchers created an immersive, interactive virtual reality (VR) driving environment designed to facilitate skill acquisition and adaptation.

The research protocol integrated an EEG activity recording system into a VR-based driving simulator to monitor mental workload, attention, and emotional states during driving tasks. The training system was composed of five interconnected subsystems: the VR driving module, which included a driving simulator (Logitech G27 Controller); the physiological data acquisition model that recorded real-time physiological responses; the EEG data acquisition model that used the Emotiv EPOC headset to track brain activity; the gaze data acquisition model that utilised the Tobii eye-tracking device to evaluate gaze fixation; and the therapist evaluation system, which provided expert assessments during and after sessions. The EEG electrodes were placed at 14 standardised sites (AF3, F7, F3, FC5, T7, P7, O1, O2, P8, T8, FC6, F4, F8, and AF4) to capture comprehensive neural data.

The study involved 16 boys aged 13–18 years (mean age 15.2), all diagnosed with ASD. The participants engaged in six one-hour sessions conducted on different days, although attendance varied: 12 teenagers completed all sessions, 1 attended four, and 3 participated in only two sessions. A monetary incentive was provided after each session. The virtual environment presented a driving simulation with six progressively challenging levels, requiring participants to operate a virtual vehicle using a steering wheel and pedal setup.

Data analysis revealed encouraging outcomes. Over 80% of the participants demonstrated positive categorisation in mental workload and attention levels, while emotional state parameters—including enjoyment, engagement, and concern—were positively evaluated at rates exceeding 75%. These results underscore the potential of integrating interface systems and complex sensory data in VR environments to enrich the interaction of individuals with ASD with their surroundings.

The research team succeeded in fully embedding an adaptable interface system into the VR environment, tailoring the system to the unique needs of each user. They conclude that such integrative systems hold significant promise for enhancing the skills and autonomy of individuals on the autism spectrum, paving the way for future advancements in adaptive technologies.

Two years after their initial study, Fan et al. [38] conducted another investigation into signal recording during driving tasks for adolescents with autism spectrum disorder (ASD) using a VR driving simulator. This iteration employed advanced software tools such as CityEngine and Autodesk Maya to create realistic driving environments and conditions. The study involved 20 participants diagnosed with ASD (19 males and 1 female) with a mean age of 15.29 years. Like the earlier research, there was no control group. Each participant completed six 60 min driving sessions on different days, with the sessions designed to mimic real-life driving challenges.

The participants were tasked with completing four driving-related activities, including turning, managing speed, interacting with other drivers, and adhering to traffic laws. The researchers developed 144 distinct driving scenarios, organised into “assignments” comprising 8 scenarios each. Each VR session included three pre-arranged assignments. Sessions were discontinued if the participants made multiple errors or deviated significantly from driving norms, prompting a restart after receiving visual and auditory feedback.

The study utilised the 14-channel wireless Emotiv EPOC headset, continuing the setup from the team’s previous research. Electrodes were placed at AF3, F3, F7, FC5, T7, P7, O1, O2, P8, and AF4, with references at P3 and P4. The EEG system operated at a bandwidth of 0.2–45 Hz with a sampling rate of 128 Hz. Physiological data were recorded using the Biopac MP 150 system, and gaze data were collected via the Tobii X120 eye tracker. These tools enabled the comprehensive monitoring of neural activity, eye movements, and physiological responses during the driving tasks.

The research team, alongside a therapist, assessed the participants’ emotional states during the sessions, focusing on levels of engagement, contentment, boredom, and frustration. They also evaluated whether the participants experienced difficulty while driving. The primary aim was to train an emotional state and workload model as a foundation for developing a new EEG-based passive brain–computer interface (BCI) system to enhance driving skills training for individuals with ASD.

These models based on EEG activations can precisely identify little interest, low pleasure, high worrying, and excessive workload for people with ASD. However, the boredom detection degree proved quite inaccurate. Interestingly, the most distinctive features for emotional impact and workload acknowledgment were recorded in the frontal areas. The EEG data analysis indicates the feasibility of EEG-based ASD personalisation of the VR-based intervention. However, additional research is necessary to determine whether the created models can accurately predict the emotional moods and mental strain of individuals with ASD, hence estimating their flow states.

This study highlighted the potential of VR driving simulators combined with BCI technology to address the unique challenges faced by individuals with ASD in acquiring driving skills. By incorporating real-time emotional and workload assessments, the research provides a foundation for adaptive systems capable of personalising training experiences to improve practical independence for this population.

Vidhusha et al. [39] investigated the potential of a cognitive virtual reality (VR) tool to address learning difficulties in children with moderate autism spectrum disorder (ASD). The study included five male participants aged 4–8 years, assessed using IQ scores and the Childhood Autism Rating Scale (CARS). Initially, the children trained with alphabet letters, numbers, and colours on flashcards, followed by similar training in a virtual classroom using a VR headset. EEG signals were recorded in both sessions to evaluate learning profiles and functional connectivity. The researchers emphasised that EEG can reveal atypical brain activity linked to cognitive challenges and brain disorders.

Using the Emotiv EPOC 14-channel headset, EEG data were transmitted via Bluetooth, with electrodes placed at AF3, AF4, F7, F3, F4, F8, FC5, FC6, T7, T8, P7, P8, O1, and O2 in the 10–20 system. The signals were sampled at 128 Hz, focusing on the alpha, beta, and gamma bands. The functional connectivity was analysed using coherence, phase locking value, correlation, and directed transfer function. These measures evaluated cognitive function and task performance during VR and flashcard sessions, providing insights into how VR impacts brain activity.

The study revealed that the VR tool outperformed flashcard-training across all parameters. The key findings concerning coherence indicated lower mean values in the alpha band during VR training, indicating increased sensitivity, while higher gamma band coherence suggested stronger connectivity across the frontal, temporal, and occipital regions. Regarding correlation, it was found that alpha band correlations were lower in VR, indicating heightened sensitivity. Beta and gamma band correlations were higher, reflecting improved attention and reduced stress during VR training.

Concerning the phase locking value, it was found that alpha band values decreased in VR, showing increased sensitivity. Variations in the beta and gamma bands were attributed to VR setup adjustments. Finally, regarding the directed transfer function, it was found that the alpha band information flow was lower, while the beta and gamma bands showed higher input–output values, reflecting enhanced cognitive processing in the frontal regions.

Overall, the results demonstrated that the VR tool improved cognitive functioning and attention more effectively than traditional flashcards. The researchers concluded that VR could serve as a valuable educational tool for children with ASD. However, they recommended expanding the VR tool with additional learning material and emphasised the need for further research to solidify these findings.

In another study, Chrisilla et al. [42] explored how children with ASD respond to VR environments using brain–computer interface (BCI) technology and whether VR could assess learning abilities. Six children aged 5–8 participated, including three with ASD and three neurotypical controls. EEG data were recorded with the Emotiv EPOC headset during immersion in a VR environment, though the VR setting was not described in detail.

The analysis focused on EEG band power, revealing that increased theta band activity in ASD participants reflected cognitive impairments, particularly in working memory. Higher delta band power suggested there was a cognitive load and its impact on neuronal processes. Amaral et al. [35] refer to the term cognitive load analysis as the assessment of the brain function during a task.

Comparing groups, the ASD group showed more activation in the occipital and frontal regions. The theta and alpha band values were higher in the ASD group, indicating a greater cognitive workload compared to the controls. Finally, both groups exhibited high delta band values, with similar trends in power reduction from the delta to gamma bands, indicating comparable cognitive processes.

The findings suggested that VR environments are effective for both neurotypical and ASD children, promoting cognitive engagement and learning. For ASD participants, the VR tool showed promise in enhancing working memory and overall cognitive function. These studies highlight the potential of VR as an innovative educational and therapeutic tool for children with ASD.

The second part of the VR-BCI protocols presentation in Table 3 concerns research protocols that used various BCI recorders such as g.Mobilab+, g.Nautilus, V-Amp, and the BrainCo FocusCalm headband, placing the active electrodes over the somatosensory cortex, the sensorimotor cortex, and the cingulate gyrus (Table 3).

The study conducted by Amaral et al. [34] focused on developing and testing an interface system integrated with virtual reality (VR) to train joint attention skills in individuals with ASD. Joint attention, an essential aspect of social interaction, involves two individuals sharing focus on a third object or person using gestures and gaze direction. This skill is often underdeveloped in individuals with ASD, particularly in situations requiring simultaneous interaction and shared goals [43].

The primary goal of the research was to identify the most effective brain–computer interface (BCI) system, combined with a head-mounted VR device, for enhancing joint attention in individuals with ASD. The team tested three distinct systems: the g.Mobilab (active dry-electrode system with wireless transmission), the g.Nautilus (active dry-electrode system using wireless technology), and the V-Amp (integrated with actiCAP Xpress electrodes and a USB 2.0 signal transmission interface via TCP/IP for remote data access).

The research included a two-phase testing approach. First, initial testing on neurotypical participants was conducted in which thirteen neurotypical individuals (aged 21–26) tested the systems to establish baselines for performance, reliability, and usability. Then, there was application to ASD individuals, in which four high-functioning male participants with ASD aged 15–21 years underwent trials based on the findings from the initial tests.

A VR environment was designed in which participants followed non-verbal social stimuli, such as head turns, to direct their attention to specific target objects. Attentional shifts were recorded using oddball detection linked to the P300 signal. The electrode placement was the same for the three set-ups, namely, C3, Cz, C4, CPz, P3, Pz, P4, and POz. The researchers hypothesised that automating responses to these social stimuli could help individuals with ASD develop joint attention capabilities.

The combination of VR and BCI technologies posed challenges, particularly in terms of achieving stable signal quality and participant comfort. The study revealed notable differences in the performance of the three systems. Namely, g.Mobilab+ and g.Nautilus outperformed V-Amp in terms of signal reliability, preparation time, and participant comfort. These systems achieved stable electrode–skin contact, leading to better data quality. V-Amp proved less effective due to discomfort caused by its electrode application, which often failed to maintain stable skin contact, impacting signal quality.

The g.Nautilus system emerged as the most suitable option, offering reliable signal acquisition, rapid preparation, and good user tolerance. In trials with ASD participants, individuals successfully followed social stimuli, interpreted activity prompts, and focused their attention on target objects. This demonstrated that the system effectively supported training in joint attention skills. However, there is need for additional clinical trials to further validate its effectiveness and scalability. This study underscores the potential of integrating VR with BCI technology to address core social deficits in ASD, paving the way for more sophisticated interventions in the future.

The study by Amaral et al. [35] introduced an immersive virtual reality (VR) protocol aimed at enhancing social attention in individuals with autism spectrum disorder (ASD). The research sought to determine whether an EEG-based brain–computer interface (BCI) system could be utilised to train social cognitive skills by analysing responses to joint attention prompts. The study combined the advantages of interactive virtual environments with the P300 brain signal’s attention-related properties to create a novel cognitive training tool.

Fifteen high-functioning subjects with ASD participated in the study. The protocol involved seven BCI sessions: four weekly sessions and three monthly follow-ups. Assessments were conducted before and after the seven sessions, with an additional follow-up six months later to evaluate the longevity of any benefits. The eye-tracking data were recorded to analyse the participants’ focus during interactions with avatars in the VR environments.

Specifically, the participants engaged with four immersive VR environments: a café with an avatar waitress, a classroom with an avatar teacher, a kiosk with an avatar shopkeeper, and a zebra crossing with an avatar on the opposite side. These environments provided a 360° perspective, including furniture and relevant objects, creating a realistic and interactive experience. The participants were tasked with identifying objects highlighted by avatar movements and responding to non-verbal cues.

Each session consisted of three phases. First, the users attended to a specific object. Then, they tracked objects selected by avatars to practice social joint attention. Finally, there was the real-time training where users responded to avatars’ non-verbal action cues while focusing on objects. Objects blinked randomly, and the participants counted the flashes. Success was indicated by objects turning green, while errors were marked by red flashes.

The immersive VR experience was delivered using an Oculus Rift Development Kit 2, and EEG signals were recorded with a g.Nautilus wireless system. Active electrodes were placed at C3, C4, P3, and POz, with a ground electrode at AFz and reference at the right ear. Eye-tracking was integrated into the headset for simultaneous data acquisition.

Although the primary goal of improving immediate response rates to joint attention cues was not achieved, the study yielded several positive outcomes. The participants exhibited improvements in mood, cognitive state, and sensory awareness. The ability to infer intentions from gaze direction and other non-verbal social attention markers also improved. Interestingly, the positive changes were maintained during the six-month follow-up, implicating sustained effects.

The stability and accuracy of the BCI system throughout the sessions reinforced its potential as a training tool for ASD. The P300 signal-based feedback system effectively helped users evaluate and adjust their attentional focus. EEG-based BCI paradigms in immersive VR environments show promise in addressing ASD symptoms. While immediate response to joint attention cues requires further refinement, the study demonstrated significant benefits in emotional and sensory domains. Future iterations of the system could enhance its ability to train social attention skills, supporting the daily lives of individuals with ASD.

The research team of Arpaia et al. [40] developed a protocol that integrates brain–computer interface (BCI) technology with augmented reality (AR) and a robotic system. This innovative approach aimed to address attention difficulties, hyperactive behaviours, and impulsivity in children with autism spectrum disorder (ASD). The system combined steady-state visual evoked potential (SSVEP)-based BCI, AR glasses, and a rehabilitation robot, providing visual and auditory feedback to users.

The BCI system consisted of two active dry electrodes (placed at FPz and Oz) and a reference electrode (on the wrist); SSVEP signal analysis, allowing for differentiation between occipital and frontal signals to reduce muscle and optical artefacts; AR glasses (Moverio BT-200) that displayed two flashing arrows (representing “move left” and “move right”); and SanBot Elf, a humanoid robot, which performed movements based on the user’s commands.

The users wore AR glasses and activated the system via an Android application. The EEG signals from the electrodes were digitised by the Olimex EEG SMT and sent to the processing unit. Then, the AR glasses displayed two visual stimuli (flashing arrows). The users had to direct their attention to a specific arrow, causing the system to process the SSVEP response and instruct the robot to turn left or right. Eye blinks controlled the robot’s movement, while intentional blinks (detected as negative EEG peaks) differentiated commands from involuntary actions. A “stop” command was issued with another intentional blink. Auditory cues from the robot confirmed successful execution of commands.

The protocol was initially tested on ten healthy volunteers, achieving an average precision of 83% for detecting SSVEP-based eye blinks. This demonstrated the system’s reliability and functionality in controlled settings. Then, the system was subsequently tested with three children aged 8–10 years, all diagnosed with ASD. Two of the participants also had comorbid ADHD and were on medication, while the third child exhibited atypical ADHD traits. The children varied in functioning levels as per the Clinical Global Impression (CGI) scale, and all exhibited challenges with attention, hyperactivity, and impulsivity.

The first child immediately wanted to try the device and completed the process. The second was hesitant at first but took part and partially completed the process because of uneasiness. The third child refused to wear the glasses and electrodes and finally tried through a customised touch screen. They did not complete the process, though. According to the research team, it is important for users with high levels of anxiety to first familiarise themselves with the system. The study showed promising results in maintaining attention among children with ASD. Despite challenges related to user anxiety and device compliance, the system’s innovative combination of BCI, AR, and robotics demonstrated its potential as a rehabilitation tool. The researchers noted the importance of tailoring the protocol to individual needs, especially for users with heightened anxiety. Further research is needed to refine the system and confirm its effectiveness in larger and more diverse ASD populations.

The research team of Lyu et al. [41] developed Eggly, an augmented reality (AR) neurofeedback game designed for preschool children with autism spectrum disorder (ASD). The game leverages an EEG headband and tablet-based AR platform to enhance attention and social skills, using mu rhythm suppression as a real-time indicator of attentional focus and social awareness.

In the game’s narrative, players help gather eggs from a farm bird. Key features include, first of all, real-time EEG-based feedback, where mu rhythm suppression is measured, with higher suppression values indicating better focus. Then, dynamic social-awareness rewards are present, as the game adapts elements such as the egg collection speed, background music tempo, and the facial expressions of characters to reward attentive behaviour. Interactive design is another key feature, since the game encourages children to regulate their attention and social skills through engaging and dynamic gameplay.

Eggly consists of three components: the BrainCo FocusCalm headband, which records real-time EEG data and is designed for comfort, making it suitable for children with ASD; a tablet App that comprises the front end of the game and is developed with an AR Kit XR Plugin and Unity XR API, offering a seamless AR experience on tablets and smartphones; and finally, a back-end server that processes EEG data and adjusts gameplay in real time. A two-way TCP channel allows data to flow between the EEG headband, tablet, and server.

The pilot testing was conducted with a six-year-old high-functioning boy with ASD at a special education centre. Feedback from this phase led to improvements in the game’s animations, sound effects, and facial expressions. The main study involved five children with ASD in a 3-week program. Each game session lasted 4–5 min, and the children were accompanied by parents or caregivers, who provided verbal encouragement as needed. The caregivers and parents were invited to complete questionnaires and participate in semi-structured interviews to share their observations and feedback. They also monitored the neurofeedback process, using recorded performance metrics to assess the child’s progress.

The game helped children improve focus and grasp spatial relationships. It also provided valuable insights into the development of AR-based gamified interventions for ASD. However, there was difficulty in precisely measuring attention spans and behaviours without complementary devices, like eye trackers. Other limitations of the study are the reliance on caregiver-reported data, which are less objective than psychometric tools, and the small sample size that restricts the reliability of the findings.

Nonetheless, Eggly demonstrated the potential of AR-based neurofeedback games as intervention tools for young children with ASD. While the study highlighted its success in fostering attention and social awareness, further research with larger, more diverse samples and additional assessment tools is needed to validate these findings.

## 5. Discussion

In this discussion part, the main conclusions and results from the literature are summarised, and their implications for our comprehension of the usefulness of VR-BCI paradigms and their potential to alleviate core symptoms in ASD are examined. It emphasises the significance of individualised sessions for young subjects with ASD and the effect of VR on cognitive processes through NFT.

Virtual reality technology and brain–computer interfaces together represent a promising new area for neuroscience research and therapeutic application. The literature conclusions are indicative of how VR-based therapies may support brain adaptation and improve cognitive functioning. According to recent research, virtual reality environments have the power to alter brain processes, activate particular brain regions, and affect a range of cognitive abilities, such as memory, attention, insight, and motor control.

Namely, engagement in therapeutic games provided in virtual reality environments seems to be effective in children on the autistic spectrum since it activates and engages them in activities [45,46,47,48], improves combined attention skills, and develops language and communication skills [19,49,50]. Additionally, virtual games seem to reinforce the development of and improvement in social skills and behaviours [20,22,25,51,52,53,54], the development of symbolic play and imagination [55], the recognition of emotions through facial expressions, and finally the training to acquire appropriate emotional responses [36,56,57,58,59].

According to these findings, VR-BCI protocols have been examined as a potential tool for improving social interaction, memory, attention, and cognitive skills in individuals with ASD. Research in this area has mainly focused on using virtual reality environments as a way to provide interactive and immersive experiences for individuals with ASD, with the hope of improving their skills and reducing anxiety. Immersive virtual reality systems have also proved safe and controlled alternatives to implementing interface technology, serving as a transition from the lab to the real world [60]. According to them, the feedback provided through these systems simulates real-life stimuli, let alone the fact that users find the whole process quite enjoyable, which is essential for intensive training protocols.

In light of our review, we examined non-invasive VR BCI protocols implemented through the electrodes’ placement on the subject’s scalp.

We observed that five research teams [36,37,38,39,42] placed the active electrodes in the same frontal (AF3, AF4, F7, F8, F3, and F4), frontal/central (FC5 and FC6), temporal (T7 and T8), parietal (P7 and P8), and occipital (O1 and O2) locations, typical of the Emotiv EPOC 14 channel BCI headsets, and in accordance with the worldwide 10–20 system [61].

These studies investigated brain–computer interface (BCI) protocols that merged virtual reality (VR) and EEG-based monitoring to improve the cognitive, social, and physical skills of people with autism spectrum disorder (ASD). These studies aimed at evaluating and training workload, memory, learning patterns, and engagement by combining real-time physiological and brain data.

The second series of studies demonstrated the potential for flexible VR therapies by utilising several EEG-based BCI settings with VR environments to analyse cognitive and emotional reactions in individuals with ASD. Every protocol used immersive or augmented reality in conjunction with EEG-based BCI to train social attention, focus, and cognitive skills in people with ASD.

Specifically, one team [35] used the C3, C4, P3, and PO2 central/parietal locations over the somatosensory cortex, one team aimed at the sensorimotor cortex to train mu rhythm suppression [41], and one team [40] placed the electrodes aiming at the cingulate gyrus. Lastly, one team [34] chose to evaluate three distinct BCI systems on the somatosensory cortex.

It is essential to note that evaluations by parents and/or caregivers [41] were added to the analysis of EEG data [35,40,41], reducing the strength of any positive outcomes. Parental observations specifically, while valuable, may introduce subjective biases into the assessment of outcomes or interpretation of results.

According to the electrodes’ placement, the protocols applied aimed at improving emotional regulation, memory attention, social skills on the frontal/central lobes, verbal recognition and language skills on the temporal lobes, visual processing and learning on the occipital lobes, and association skills and attention on the parietal lobes. Attention skills were also trained on the cingulate gyrus and on the somatosensory cortex, whereas social and communication skills were also trained on the sensorimotor cortex.

Consequently, the studies presented in this paper have shown that such BCI-VR systems can provide individuals with ASD with a safe and controlled environment, where they can practice social interactions such as eye contact and conversation [36]. They have also shown that these systems can help improve communication and emotional regulation for individuals with ASD [35]. Moreover, neurofeedback training in ASD has helped children generalise learned behaviours to everyday situations, such as driving [37,38]. As far as joint attention training is concerned, which further hinders the development of social and language skills [43], the studies of Amaral et al. [34,35] proved partially successful. The users also learned to direct their attention to target objects and to interpret someone’s intentions through gaze direction.

The working memory of the users also improved [42], and maintaining attention was successful throughout the research of Arpaia et al. [40]. Lyu et al. [41] managed to train social and communication skills. Additionally, positive changes in the cognitive state of the subjects were confirmed, in the research of Amaral et al. [35], Vidhusha et al. [39], and Chrisilla et al. [42], as the users were keener on learning new material and making decisions. Finally, sensory awareness was enhanced in Amaral et al. [35], while subjects in Lyu et al. [41] trained their social attention skills and also managed to perceive objects from different angles.

Nonetheless, there are certain limitations in all studies, which weaken the positive results of the research protocols. First of all, we should mention the absence of control groups in most papers (seven out of nine) and the rather small number of participants in the target groups trained. Namely, the small sample size used in most studies limits the generalisability and statistical power of the results, making it difficult to draw robust conclusions about the effectiveness of the protocols. Additionally, it is difficult to ascertain whether improvements in core ASD symptoms are attributable to the VR intervention or other outside influences in the absence of a control group.

Another limitation common in all studies is the absence of follow-up assessment. Only one study [35] included an evaluation after six months to check the sustainability of the outcomes. Certainly, the lack of follow-up data raises concerns regarding the sustainability and longevity of the advantages that have been observed.

Proceeding with the limitations of the studies, it is useful to mention that five studies on ASD included male participants [34,36,37,38,39]. One study also included a female participant [38]. In the rest of the studies, the gender of the participants was not specified [35,40,41,42]. The absence of female participants limits the applicability of the findings to girls with ASD, who may exhibit different learning profiles and cognitive responses. However, it is worth noting that the prevalence of ASD in boys is four times higher than in girls [62]. Although the over-representation of boys may be explained by this, the studies’ lack of female participants is still a disadvantage.

Another limitation of the studies we examined is that they predominately focus on high-functioning ASD individuals, underrepresenting those with severe symptoms or comorbidities. Concerning ASD severity, only in one study [39] were the subjects of medium-level ASD. However, it is worth mentioning that across all metrics, the VR tool training performed better than the traditional flashcard training.

Regarding comorbidities, the three individuals in a single study [40] displayed signs of ADHD or comorbid ADHD. Not all of them managed to complete the training process though, as described in Section 4, which is indicative of the need to adjust protocols when such comorbidities occur in order to diminish problems of distraction, hyperactivity, and impulsivity [63]. Unfortunately, the study did not account for how these comorbid conditions might influence the system’s outcomes, potentially confounding the results.

We point out a few more paper-specific constraints in an effort to be more analytical and prevent any potential bias.

In one study [36], the target group showed only a 3% improvement in eye contact, with no statistically significant differences in other metrics. The control group did not engage in visual analysis, feedback protocols, or face-activity tasks. The modest results raise questions about the practical impact of the intervention and its potential scalability.

In another study [34], it was not specified whether the training was conducted over multiple sessions. The study also highlights issues with achieving stable signal quality across the tested systems, particularly with the V-Amp device. These technical limitations may impact the reliability and consistency of the results, as poor signal quality can compromise data accuracy.

Participation ranged from two to six sessions in another study [37], with some participants failing to complete the full training. Uneven exposure to the protocol introduces variability that may affect the consistency and validity of the outcomes. Moreover, the limited duration may not be sufficient to develop or assess long-term driving skills or their application in real-world scenarios. Additionally, the participants were given monetary rewards for attending sessions. This could influence participation and engagement, raising questions about whether observed improvements stemmed from intrinsic motivation or external incentives.

Similarly, the protocol allowed for session restarts if participants struggled significantly [38]. Restarting sessions may affect data consistency, making it harder to evaluate participant progress over time. Moreover, the EEG model showed poor accuracy in detecting boredom and the scientists focused on specific emotional and cognitive states, overlooking other factors that significantly influence driving ability and performance in individuals with ASD.

The study by Amaral et al. [35] demonstrated the potential of a VR-BCI system for enhancing social cognitive skills in individuals with ASD, with notable improvements in mood, cognitive state, and sensory awareness. However, the inability to achieve the primary goal of improving immediate joint attention responses comprises an area for improvement.

In a single study [42], we noticed the absence of a clear description of the VR environment. Therefore, it is difficult to assess the specific factors that contributed to the observed outcomes, limiting reproducibility and practical application. The study also primarily examined EEG band power (theta, delta, and alpha bands) as indicators of cognitive workload and engagement. The analysis could also have addressed cognitive domains such as problem-solving, language skills, or attention span, which could provide a better understanding of VR impact.

We noticed that some children experienced difficulty using the VR-BCI system due to anxiety or device discomfort [40]. The usability of the protocol may be limited for individuals with heightened sensory sensitivities or anxiety [64,65], reducing its applicability across the ASD population.

Finally, despite its innovative approach, one study [41] did not incorporate additional devices, such as eye trackers, to validate attention spans or behaviours.

## 6. Conclusions

Our systematic review concludes that the combination of brain–computer interfaces and virtual reality provides a realistic solution to enhance the cognitive, social, and emotional abilities of young people with autism spectrum disorder. The reviewed studies highlight the potential of VR-BCI systems in training attention, memory, social interactions, and emotional regulation while also offering a safe and controlled environment for learning. However, limitations such as small sample sizes, the lack of control groups, limited follow-up assessments, and the underrepresentation of female participants and individuals with severe ASD symptoms restrict the generalisability of the findings. The reliability of results is further impacted by issues with signal quality, system usability, and training procedure variability. Despite these constraints, the initial outcomes are encouraging, suggesting that with further refinement and larger-scale studies, VR-BCI systems could become valuable tools for ASD intervention, providing personalised experiences that address the unique needs of young individuals on the spectrum.

## 7. Future Directions and Interdisciplinary Collaboration

It is necessary to note that more research is needed to fully understand the potential benefits and limitations of BCI-VR in ASD treatment and to determine the most effective protocols for implementation. The field is still relatively new, and more protocols are needed to solidify the long-term impact of BCI-VR on school-age individuals with ASD since the majority of the research does not involve follow-up studies. Moreover, we believe that well-matched control groups need to be incorporated in this kind of research to strengthen causal claims about the effectiveness of VR-BCI protocols.

Future studies could also focus on expanding and redefining BCI paradigms, making VR interventions more user-friendly to ASD subjects and examining the long-term possibilities of VR-BCI-enhanced treatments using AI for cognitive and executive functioning management [66]. Moreover, these treatments could appeal to a more diverse range of ASD participants to better understand the interventions’ applicability across the spectrum.

Finally, we believe that interdisciplinary cooperation among neuroscientists, engineers, parents, caregivers, and physicians is essential to advancing the field and resolving any potential clinical, technical, or even ethical issues.

BCI-VR surely holds promise as a tool for improving social and cognitive skills in individuals with ASD, but more research will establish the benefits and the limitations. The combination of BCIs and VR paves new ways to address the main symptoms of ASD, marking a paradigm change in ASD interventions. Ongoing research and development could lead to new discoveries in the field of neuroscience and change our understanding and approach to treating individuals with ASD.

## Figures and Tables

**Table 1:** Overview of the studies included for analysis.

Author/Reference	Journal	Year	Study Design	Country	Outcome Assessment	Contribution	Limitations
Bekele et al. [36]	*IEEE virtual reality (VR)*	2016	Usability Study	USA	SCQ, SRS, NEPSY, and parents	Examines the integration of peripheral data to improve emotional and social deficits in ASD via a multimodal system.	Sample size, absence of control group design, no long-term follow-up, minimal improvement, parental assessment
Amaral et al. [34]	*Journal of neuroscience methods*	2017	Feasibility Study	Portugal	Non-specified	Explores three different combined devices to train joint attention in subjects with ASD through the use of social stimuli.	Sample size, trial duration, challenges with signal stability, no long-term follow-up
Fan et al. [37]	37th Annual International Conference of the IEEE Engineering in Medicine and Biology Society (EMBC)	2015	Feasibility Study	USA	Expert therapist	Discusses the integration of an EEG activity recording interface system into a VR-based driving system to reinforce the independence of ASD individuals in areas of daily lives, such as driving.	Sample size, variability in session attendance, no control group, no long-term follow-up, therapist evaluation
Fan et al. [38]	*IEEE Transactions on Biomedical Engineering*	2018	Feasibility Study	USA	Expert therapist	Aimed to train the emotional state and workload of people with ASD so as to develop a new EEG BCI system to regulate driving skills training.	No control group, no long-term follow-up, variability in participant exposure, therapist evaluation
Amaral et al. [35]	*Frontiers in neuroscience*	2018	Feasibility Study	Portugal	POMS, JAAT, ATEC, and VABS	Attempted to develop a cognitive training tool for ASD, combining the benefits of interactive virtual worlds with the attention-related properties of P300 brain signals.	No control group, lack of immediate improvement in joint attention
Vidhusha et al. [39]	IEEE 18th International Conference on Cognitive Informatics & Cognitive Computing	2019	Feasibility Study	India	CARS	Examined whether a cognitive VR tool would improve the learning difficulties of children with a medium level of ASD.	Sample size, no control group, no long-term follow-up
Chrisilla et al. [42]	Sixth International Conference on Bio Signals, Images, and Instrumentation (IC BSII)	2020	Quasi-experimental Trial	India, USA	Non-specified	Examines VR-BCI technology as a useful educational tool, able to improve ASD children’s working memory and cognitive abilities.	Sample size, short-term study, lack of detailed VR environment description, no detailed analysis of C.G. performance
Arpaia et al. [40]	IEEE International Symposium on Medical Measurements and Applications	2020	Clinical Case Study	Italy	CGI, ADOS2, LEITER-R, and CARS2-ST	Integrated an SSVEP-based BCI into an AR platform, applied through a rehabilitation robot, providing visual and auditory feedback to the user and aiming at addressing difficulties in attention and behaviour.	Sample size, limited testing on ASD, no control group, no long-term follow-up, undefined test duration, device compliance and anxiety challenges
Lyu et al. [41]	Proceedings of the ACM on Interactive, Mobile, Wearable and Ubiquitous Technologies	2023	Field Study	Canada	Parents and caregivers	Examined whether an EEG data real-time recorder and a tablet app presenting a game in augmented reality would enhance brain function in children with ASD, modifying and regulating their attention.	Sample size, no long-term follow-up, no control group, caregiver and parent reported data

**Table 2:** BCI systems with identical configurations.

Author/Title	BCI Recording/Protocol	Participants/Age	NFT Sessions	Brain Area/Active Electrodes
Bekele et al. [36]: “Multimodal adaptive social interaction in virtual environment (MASI-VR) for children with Autism spectrum disorders (ASD).”	Emotiv EPOC, Tobii X120 eye tracker, multimodal protocol: gaze scanning, analysis and adaptation, and interactive model of structured Q/A	TG: *n* = 6 (M = 15.77) CG: *n* = 6 (M = 15.20)	3–5 sessions, 60’ each	Frontal AF3, AF4, F7, F8, F3, F4; frontal/central FC5, FC6; temporal T7, T8; parietal P7, P8; and occipital O1, O2
Fan et al. [37]: “A Step towards EEG-based brain computer interface for autism intervention.”	Emotiv EPOC, Tobii X120 eye tracker, VR Driving Module/Logitech G27 Controller	TG: *n* = 16 (M = 15.24) CG: -	6 different sessions, 60’ each	Frontal AF3, AF4, F7, F8, F3, F4; frontal/central FC5, FC6; temporal T7, T8; parietal P7, P8; and occipital O1, O2
Fan et al. [38]: “EEG-Based Affect and Workload Recognition in a Virtual Driving Environment for ASD Intervention.”	Emotiv EPOC, Tobii X120 eye tracker, integration of complex sensory data, interactive–immersive environment	TG: *n* = 20 (M= 15.29) CG: -	6 different sessions, 60’ each	Frontal/central AF3, AF4, F3, F7, FC5; temporal, T7; parietal, P7, P8; occipital, O1, O2
Vidhusha et al. [39]: “Cognitive attention in autism using virtual reality learning tool.”	Emotiv EPOC, VR classroom training with letters, numbers, and colours	TG: *n* = 5 (4–8) CG: -	Unstated training period	Frontal AF3, AF4, F7, F8, F3, F4; frontal/central FC5, FC6; temporal T7, T8; parietal P7, P8; and occipital O1, O2
Chrisilla et al. [42]: “Effect of virtual reality on the EEG sub-band frequency powers of autistic and control groups.”	Emotiv EPOC, cognitive tool	TG: *n* = 3 (5–8) CG: *n* = 3 (5–8)	Unstated training period	Frontal AF3, AF4, F7, F8, F3, F4; frontal/central FC5, FC6; temporal T7, T8; parietal P7, P8; and occipital O1, O2
		TG = target group CG = control group		

**Table 3:** BCI systems with various configurations.

Author/Title	BCI Recording/Protocol	Participants/Age	NFT Sessions	Brain Area/Active Electrodes
Amaral et al. [34]: “A novel brain computer interface for classification of social joint attention in autism and comparison of 3 experimental setups: a feasibility study.”	Ρ300 signal, g.Mobilab+, g.Nautilus, V-Amp	TG: *n* = 4 (M = 18.8) CG: -	Unstated training period	C3, Cz, C4, CPz, P3, Pz, P4, and POz (somatosensory cortex)
Amaral et al. [35]: “A feasibility clinical trial to improve social attention in autistic spectrum disorder (ASD) using a brain computer interface.”	g.Nautilus, P300 signals, Eye-Tracking HMD package, VR settings training, follow-up assessment	TG: *n* = 15 (M = 22.2) CG: -	7 sessions (4 weekly, 3 monthly afterwards)	C3, C4, P3, and POz (somatosensory cortex)
Arpaia et al. [40]: “Robotic autism rehabilitation by wearable brain-computer interface and augmented reality.”	SSVEP-based BCI into an AR platform, rehabilitation robot, AR smart glasses	TG: *n* = 3 (8–10) CG: -	Unstated training period	FPz and Oz (cingulate gyrus)
Lyu et al. [41]: “Eggly: Designing Mobile Augmented Reality Neurofeedback Training Games for Children with Autism Spectrum Disorder.”	EEG data real-time recorder and a tablet app, BrainCo FocusCalm headband	TG: *n* = 5(3–6) CG: -	3-week program, sessions 4–5 min. each	mu rhythm suppression (sensorimotor cortex)
		TG = target group CG = control group		

## Data Availability

Not applicable.

## References

[1] American Psychiatric Association (2013). Diagnostic and Statistical Manual of Mental Disorders.

[2] World Health Organization, 2019 ICD-11: 6A02—Autism Spectrum Disorder. https://icd.who.int/en.

[3] Billstendt E., Gillberg C., Gillberg C. (2005). Autism after adolescence: Population-based 13-to 22-year follow-up study of 120 individuals with autism diagnosed in childhood. J. Autism Dev. Disord..

[4] Farley M.A., McMahon W.M., Fombonne E., Jenson W.R., Miller J., Gardner M., Block H., Pingree C.B., Ritvo E.R., Ritvo R.A. (2009). Twenty-year outcome for individuals with autism and average or near-average cognitive abilities. Autism Res..

[5] Van Kokswijk J., Van Hulle M. Self-adaptive BCI as service-oriented information system for patients with communication disabilities. Proceedings of the 4th International Conference on New Trends in Information Science and Service Science.

[6] Mattout J. (2012). Brain-Computer Interfaces: A Neuroscience Paradigm of Social Interaction? A Matter of Perspective. Front. Hum. Neurosci..

[7] Wolpaw J.R., Birbaumer N., McFarland D.J., Pfurtscheller G., Vaughan T.M. (2002). Brain–computer interfaces for communication and control. Clin. Neurophysiol..

[8] Vuckovic A., Pineda J.A., LaMarca K., Gupta D., Guger C. (2014). Interaction of BCI with the underlying neurological conditions in patients: Pros and cons. Front. Neuroeng..

[9] Wolpaw J.R., Wolpaw E.W., Wolpaw J.R., Wolpaw E.W. (2012). Brain-computer interfaces: Something new under the sun. Brain Computer Interfaces: Principles and Practice.

[10] Renard Y., Lotte F., Gibert G., Congedo M., Maby E., Delannoy V., Bertrand O., Lécuyer A. (2010). Openvibe: An open-source software platform to design, test, and use brain–computer interfaces in real and virtual environments. Presence.

[11] Lotte F. (2015). Signal processing approaches to minimize or suppress calibration time in oscillatory activity-based brain–computer interfaces. Proc. IEEE.

[12] Drigas A., Vlachou J.A. (2016). Information and communication technologies (ICTs) and autistic spectrum disorders (ASD). Int. J. Recent Contrib. Eng. Sci. IT.

[13] Alexopoulou A., Batsou A., Drigas A.S. (2019). Effectiveness of Assessment, Diagnostic and Intervention ICT Tools for Children and Adolescents with ADHD. Int. J. Recent Contrib. Eng. Sci. IT.

[14] Grynszpan O., Weiss P.L., Perez-Diaz F., Gal E. (2013). Innovative technology-based interventions for autism spectrum disorders: A meta-analysis. Autism.

[15] Wainer A.L., Ingersoll B.R. (2011). The use of innovative computer technology for teaching social communication to individuals with autism spectrum disorders. Res. Autism Spectr. Disord..

[16] Bharatharaj J., Kumar S.S. Considerations in Autism therapy using robotics. Proceedings of the 2013 Fourth International Conference on Computing, Communications and Networking Technologies (ICCCNT).

[17] Ozonoff S. (1995). Reliability and validity of the Wisconsin card sorting test in studies of autism. Neuropsychology.

[18] Tseng R.Y., Do E.Y.L. (2011). The role of information and computer technology for children with autism spectrum disorder and the facial expression wonderland (FeW). Int. J. Comput. Models Algorithms Med. (IJCMAM).

[19] Bellani M., Fornasari L., Chittaro L., Brambilla P. (2011). Virtual reality in autism: State of the art. Epidemiol. Psychiatr. Sci..

[20] Mitchell P., Parsons S., Leonard A. (2007). Using virtual environments for teaching social understanding to 6 adolescents with autistic spectrum disorders. J. Autism Dev. Disord..

[21] Teo W.P., Muthalib M., Yamin S., Hendy A.M., Bramstedt K., Kotsopoulos E., Perrey S., Ayaz H. (2016). Does a combination of virtual reality, neuromodulation and neuroimaging provide a comprehensive platform for neurorehabilitation?–a narrative review of the literature. Front. Hum. Neurosci..

[22] Cheng Y., Ye J. (2010). Exploring the social competence of students with autism spectrum conditions in a collaborative virtual learning environment–The pilot study. Comput. Educ..

[23] Jarrold W., Mundy P., Gwaltney M., Bailenson J., Hatt N., McIntyre N., Kim K., Solomon M., Novotny S., Swain L. (2010). Social attention in a virtual public speaking task in higher functioning children with autism. Autism Res..

[24] Kandalaft M.R., Didehbani N., Krawczyk D.C., Allen T.T., Chapman S.B. (2013). Virtual reality social cognition training for young adults with high-functioning autism. J. Autism Dev. Disord..

[25] Didehbani N., Allen T., Kandalaft M., Krawczyk D., Chapman S. (2016). Virtual reality social cognition training for children with high functioning autism. Comput. Hum. Behav..

[26] Wang M., Anagnostou E. (2014). Virtual reality as treatment tool for children with autism. Comprehensive Guide to Autism.

[27] Parsons S., Mitchell P. (2002). The potential of virtual reality in social skills training for people with autistic spectrum disorders. J. Intellect. Disabil. Res..

[28] Babu P.R.K., Oza P., Lahiri U. (2017). Gaze-sensitive virtual reality based social communication platform for individuals with autism. IEEE Trans. Affect. Comput..

[29] Teo A.J., Mishra A., Park I., Kim Y.J., Park W.T., Yoon Y.J. (2016). Polymeric biomaterials for medical implants and devices. ACS Biomater. Sci. Eng..

[30] Williams R.M., Gilbert J.E. (2020). Perseverations of the academy: A survey of wearable technologies applied to autism intervention. Int. J. Hum.-Comput. Stud..

[31] Lorenzo G., Lledó A., Pomares J., Roig R. (2016). Design and application of an immersive virtual reality system to enhance emotional skills for children with autism spectrum disorders. Comput. Educ..

[32] Beach J., Wendt J. (2014). Social Interaction development through immersive virtual environments. Proceedings of the International Conference on Education Technologies and Computers.

[33] Siano M., Pellegrino L., Casadio M., Summa S., Garbarino E., Rossi V., Dall’Agata D., Sanguineti V. (2015). Natural interfaces and virtual environments for the acquisition of street crossing and path following skills in adults with Autism Spectrum Disorders: A feasibility study. J. NeuroEngineering Rehabil..

[34] Amaral C.P., Simões M.A., Mouga S., Andrade J., Castelo-Branco M. (2017). A novel brain computer interface for classification of social joint attention in autism and comparison of 3 experimental setups: A feasibility study. J. Neurosci. Methods.

[35] Amaral C., Mouga S., Simões M., Pereira H.C., Bernardino I., Quental H., Playle R., McNamara R., Oliveira G., Castelo-Branco M. (2018). A feasibility clinical trial to improve social attention in autistic spectrum disorder (ASD) using a brain computer interface. Front. Neurosci..

[36] Bekele E., Wade J., Bian D., Fan J., Swanson A., Warren Z., Sarkar N. (2016). Multimodal adaptive social interaction in virtual environment (MASI-VR) for children with Autism spectrum disorders (ASD). 2016 IEEE Virtual Reality (VR).

[37] Fan J., Wade J.W., Bian D., Key A.P., Warren Z.E., Mion L.C., Sarkar N. A Step towards EEG-based brain computer inter-face for autism intervention. Proceedings of the 37th Annual International Conference of the IEEE Engineering in Medicine and Biology Society (EMBC).

[38] Fan J., Wade J.W., Key A.P., Warren Z.E., Sarkar N. (2018). EEG-Based Affect and Workload Recognition in a Virtual Driving En-vironment for ASD Intervention. IEEE Trans. Biomed. Eng..

[39] Vidhusha S., Divya B., Kavitha A., Narayanan R.V., Yaamini D. Cognitive attention in autism using virtual reality learning tool. Proceedings of the 2019 IEEE 18th International Conference on Cognitive Informatics & Cognitive Computing (ICCI* CC).

[40] Arpaia P., Bravaccio C., Corrado G., Duraccio L., Moccaldi N., Rossi S. Robotic autism rehabilitation by wearable brain-computer interface and augmented reality. Proceedings of the 2020 IEEE International Symposium on Medical Measurements and Applications (MeMeA).

[41] Lyu Y., An P., Xiao Y., Zhang Z., Zhang H., Katsuragawa K., Zhao J. (2023). Eggly: Designing Mobile Augmented Reality Neurofeedback Training Games for Children with Autism Spectrum Disorder. Proc. ACM Interact. Mob. Wearable Ubiquitous Technol..

[42] Chrisilla S., Masciantonio A., Divya B., Vidhusha S., Kavitha A. Effect of virtual reality on the EEG sub-band frequency powers of autistic and control groups. Proceedings of the 2020 Sixth International Conference on Bio Signals, Images, and Instrumentation (ICBSII).

[43] Charman T. (2003). Why is joint attention a pivotal skill in autism?. Philos. Trans. R. Soc. London. Ser. B Biol. Sci..

[44] Zhu H., Zhao M., Sun Y. A Prototype System for Autism Rehabilitation Based on Broken Mirror Theory. In International Conference on Advances in Energy, Environment and Chemical Engineering; Atlantis Press: 2015; pp. 312–315.

[45] Larrain-Valenzuela J., Zamorano F., Soto-Icaza P., Carrasco X., Herrera C., Daiber F., Aboitiz F., Billeke P. (2017). Theta and alpha oscillation impairments in autistic spectrum disorder reflect working memory deficit. Sci. Rep..

[46] Mineo B.A., Ziegler W., Gill S., Salkin D. (2009). Engagement with electronic screen media among students with autism spectrum disorders. J. Autism Dev. Disord..

[47] Chaidi I., Drigas A. (2020). Digital games & special education. Tech. Soc. Sci. J..

[48] Stathopoulou A., Karabatzaki Z., Tsiros D., Katsantoni S., Drigas A. (2019). Mobile apps the educational solution for autistic students in secondary education. Int. J. Interact. Mob. Technol..

[49] Simões M., Carvalho P., Castelo-Branco M. Virtual reality and brain-computer interface for joint-attention training in autism. Proceedings of the 13th International Conference on Disability, Virtual Reality & Associated Technologies.

[50] Drigas A., Petrova A. (2014). ICTs in speech and language therapy. Int. J. Eng. Pedagog..

[51] Bernardini S., Porayska-Pomsta K., Smith T.J. (2013). ECHOES: An intelligent serious game for fostering social communication in children with autism. Inf. Sci..

[52] Kuriakose S., Lahiri U. (2016). Design of a physiology-sensitive VR-based social communication platform for children with autism. IEEE Trans. Neural Syst. Rehabil. Eng..

[53] Drigas A., Mitsea E., Skianis C. (2022). Virtual Reality and Metacognition Training Techniques for Learning Disabilities. Sustainability.

[54] Halabi O., Abou El-Seoud S., Alja’am J., Alpona H., Al-Hemadi M., Al-Hassan D. (2017). Design of immersive virtual reality system to improve communication skills in individuals with autism. Int. J. Emerg. Technol. Learn..

[55] Herrera G., Alcantud F., Jordan R., Blanquer A., Labajo G., De Pablo C. (2008). Development of symbolic play through the use of virtual reality tools in children with autistic spectrum disorders: Two case studies. Autism.

[56] Wallace S., Parsons S., Westbury A., White K., White K., Bailey A. (2010). Sense of presence and atypical social judgments in immersive virtual environments: Response of adolescents with autistic spectrum disorder. Autism Int. J. Res. Pract..

[57] Deriso D., Susskind J., Krieger L., Bartlett M. (2012). Emotion mirror: A novel intervention for autism based on real-time expression recognition. Proceedings of the Computer Vision–ECCV 2012. Workshops and Demonstrations.

[58] Harms M.M., Martin A., Wallace G.L. (2010). Facial Emotion Recognition in Autism Spectrum Disorders: A review of Behavioral and Neuroimaging Studies. Neuropsychol. Rev..

[59] Drigas A., Sideraki A. (2021). Emotional Intelligence in Autism. Tech. Soc. Sci. J..

[60] Friedman D., Leeb R., Pfurtscheller G., Slater M. (2010). Human–computer interface issues in controlling virtual reality with brain–computer interface. Hum. Comput. Interact..

[61] Demos J.N. (2005). Getting Started with Neurofeedback.

[62] Loomes R., Hull L., Mandy W.P.L. (2017). What is the male-to-female ratio in autism spectrum disorder? A systematic review and meta-analysis. J. Am. Acad. Child Adolesc. Psychiatry.

[63] Doulou A., Pergantis P., Drigas A., Skianis C. (2025). Managing ADHD Symptoms in Children Through the Use of Various Technology-Driven Serious Games: A Systematic Review. Multimodal Technol. Interact..

[64] Pergantis P., Drigas A. (2023). Assistive Technology for Autism Spectrum Disorder Children That Experiences Stress and Anxiety. Braz. J. Sci..

[65] Pergantis P., Drigas A. (2023). Sensory Integration Therapy as Enabler for Developing Emotional Intelligence in Children with Autism Spectrum Disorder and the ICT’s Role. Braz. J. Sci..

[66] Pergantis P., Bamicha V., Skianis C., Drigas A. (2025). AI Chatbots and Cognitive Control: Enhancing Executive Functions Through Chatbot Interactions: A Systematic Review. Brain Sci..

